# Psoriasis comorbidities: complications and benefits of immunobiological treatment*

**DOI:** 10.1590/abd1806-4841.20165080

**Published:** 2016

**Authors:** André Vicente Esteves de Carvalho, Ricardo Romiti, Cacilda da Silva Souza, Renato Soriani Paschoal, Laura de Mattos Milman, Luana Pizarro Meneghello

**Affiliations:** 1 Santa Casa de Misericórdia de Porto Alegre – Porto Alegre (RS), Brazil; 2 Universidade de São Paulo (USP) – São Paulo (SP), Brazil; 3 Private clinic – São Paulo (SP), Brazil

**Keywords:** Comorbidity, Metabolic Syndrome X, Psoriasis, Tumor necrosis factor-alpha

## Abstract

During the last decade, different studies have converged to evidence the high prevalence of comorbidities in subjects with psoriasis. Although a causal relation has not been fully elucidated, genetic relation, inflammatory pathways and/or common environmental factors appear to be underlying the development of psoriasis and the metabolic comorbidities. The concept of psoriasis as a systemic disease directed the attention of the scientific community in order to investigate the extent to which therapeutic interventions influence the onset and evolution of the most prevalent comorbidities in patients with psoriasis. This study presents scientific evidence of the influence of immunobiological treatments for psoriasis available in Brazil (infliximab, adalimumab, etanercept and ustekinumab) on the main comorbidities related to psoriasis. It highlights the importance of the inflammatory burden on the clinical outcome of patients, not only on disease activity, but also on the comorbidities. In this sense, systemic treatments, whether immunobiologicals or classic, can play a critical role to effectively control the inflammatory burden in psoriatic patients.

## INTRODUCTION

During the last decade, different studies have converged to evidence the high prevalence of metabolic syndrome in patients with psoriasis. Although a causal relation has not been fully elucidated, genetic relation, inflammatory pathways and/or common environmental factors appear to be underlying to the development of psoriasis and metabolic comorbidities. These phenotypically distinct disorders would share similar pathophysiological phenomena: chronic inflammation, angiogenesis, oxidative stress, and selected genes and susceptibility loci. ^1,2^

Proinflammatory cytokines and other factors overproduced in psoriasis contribute to the increased risk of developing metabolic syndrome (MS) and cardiovascular disease. The activation and expansion of Th-1 and Th-17 cells would be responsible for the high production of inflammatory cytokines, especially TNF-alpha, IFN-gamma, IL-1, IL-2, IL-6, IL-8 and IL-17, with effects on several processes, such as angiogenesis, adipogenesis, insulin signaling, lipid metabolism and immune cell traffic. Metabolic aspects of chronic inflammation Th-1/Th-17 in psoriasis would imply significant impact on other conditions (obesity, diabetes and atherosclerosis) in a reciprocal relation of aggravation and predisposition. ^2,3^

Systematic reviews and meta-analyzes of observational studies have shown the association of psoriasis to increased prevalence and incidence of MS, as well as its individual components: obesity, dyslipidemia, diabetes mellitus and hypertension. In addition, severe psoriasis patients compared with those with the mild form of the disease presented higher chances for the development of MS and its components. ^4,5^

The concept of psoriasis as a systemic disease directed the attention of the scientific community in order to investigate the extent to which therapeutic interventions influence the initiation and evolution of the prevalent comorbidities in patients with psoriasis. Different studies and registries have investigated the role of the different treatments of psoriasis on comorbidities.

Researching the terms "*psoriasis AND comorbidities*" on available databases, an extensive list of diseases is found, ranging from erectile dysfunction to bronchial asthma. However, the most commonly comorbid conditions related to psoriasis are:

1. Metabolic syndrome and its components:
  **1a.** Central obesity
  **1b.** Atherogenic dyslipidemia
  **1c.** Systemic arterial hypertension
  **1d.** Insulin resistance
2. Cardiovascular disease
3. Nonalcoholic steatohepatitis

We will discuss below the scientific evidence of the influence of immunobiological treatments for psoriasis available in Brazil (infliximab, adalimumab, etanercept and ustekinumab) on the main comorbidities related to psoriasis.

## IMMUNOBIOLOGICALS

Immunobiologicals are proteinaceous drugs, obtained through modern biotechnological techniques that interfere in a specifically and timely manner in the immune system, blocking or stimulating one or more pathways of the immune response. Infliximab, etanercept and adalimumab are drugs that block the action of tumor necrosis factor alpha (TNF-α) while the ustekinumab is a drug that blocks the p40 subunit of the receptors of interleukin 12 and 23, inhibiting their action.

Despite differences in the mechanism of action, the response to the use of immunobiologicals includes a reduction in inflammatory burden observed in diseases such as psoriasis, rheumatoid arthritis, Crohn's disease and many other immune-mediated inflammatory diseases. In turn, this inflammatory burden also appears to relate to the occurrence of different comorbidities associated with these diseases. Consequently, it is worth considering that if the immunobiologicals decrease the inflammatory burden responsible for comorbidities, its use could inhibit their onset or aggravation. Moreover, immunobiologicals, like any other medication, have adverse events that can negatively interfere with these comorbidities.

## 1. METABOLIC SYNDROME AND PSORIASIS

Metabolic syndrome (MS) is a complex entity represented by a set of cardiovascular risk factors usually related to insulin resistance and central adiposity. Among the related factors are hypertension, abdominal obesity, dyslipidemia and glucose intolerance that, when associated, are demonstrably related to the higher incidence of cardiovascular risk. ^6^

Various study groups have developed criteria for the diagnosis of MS. The definition of the National Cholesterol Education Program - Adult Treatment Panel III (NCEP-ATP III) is the most widely used and the one recommended by the Brazilian Guidelines for Diagnosis and Treatment of MS. ^7,8^ According to the NCEP-ATP III, MS represents the combination of at least three of the following five components: changes in waist circumference, blood pressure, triglycerides, HDL-cholesterol and fasting glucose levels.

A meta-analysis demonstrated that patients with psoriasis have a higher prevalence of metabolic syndrome (from 14% to 40%) compared with the general population, and the greater the severity, the strongest is the disease association. ^9,10^

Despite the high frequency of smoking and increased alcohol consumption among individuals with psoriasis, the association between psoriasis and MS has been shown independent. Multivariate analysis models adjusted for age, gender and smoking condition of psoriasis patients showed that psoriasis was consistently associated with MS. ^11^ There are also indications that the severity of psoriasis has direct relation to the increased risk for the development of MS.^12^ The increased prevalence of MS and its components seem to also be present in children with psoriasis.^13,14^

### 1a. INSULIN RESISTANCE AND DIABETES MELLITUS

Insulin resistance was found in non-obese patients with psoriasis and was correlated with PASI (Psoriasis Area and Severity Index). Many studies have found increased risk of diabetes mellitus (DM) in patients with psoriasis.^10^

In patients with psoriatic arthritis, the use of anti-TNF-α was associated with reduced risk of developing DM (OR = 0.62) compared with the use of other disease modifying drugs (except methotrexate). ^15^

#### Infliximab

There are no randomized placebo-controlled trials or even case series evaluating the relation between the use of infliximab and improvement or worsening of glucose tolerance in patients with psoriasis.^16^

In animal models, there is evidence that the administration of infliximab can delay the onset of glucose intolerance and metabolic syndrome, during the induction of obesity in mice. The treated animals showed marked decrease in fasting glucose levels and steady decline in blood glucose levels during glucose tolerance test.^17^

Some uncontrolled studies of patients with rheumatoid arthritis and ankylosing spondylitis show a potential benefit of infliximab in increasing insulin sensitivity.^18-20^

In Crohn's disease, in the analysis of a series of cases, there appeared to be an improvement in glycemic indices in patients treated with infliximab during the maintenance phase of treatment for a year or more. ^21^

Wascher *et al.*, on the other hand, in a randomized, placebo-controlled trial, compared the effect of infusion of infliximab during 32 weeks on glucose tolerance in obese men, otherwise healthy, and concluded that there is no influence of medication on glucose levels or on insulin sensitivity. It is noteworthy, however, that the study randomized only nine patients, five in the group receiving infliximab and four in the placebo group, which is an unrepresentative sample of the population under study. ^22^

Finally, there are reports of patients with rheumatoid arthritis who presented abrupt and symptomatic changes in blood glycemic levels (hypoglycemia and hyperglycemia) after the use of drugs that block TNF-α.^23,24^

#### Etanercept

Two studies examined the influence of treatment with etanercept on insulin resistance in patients with psoriasis. In a randomized, placebo-controlled study, Martinez-Abundis *et al.* studied the effect of infusion of 50 mg etanercept weekly or placebo in 12 patients with psoriasis and found no alteration in insulin secretion or insulin sensitivity after two weeks of treatment. Arguments against this study can be raised to the extent that the sample is modest and the follow-up is too short.^25^ Marra *et al.* evaluated, in a case series, nine patients with psoriasis who used etanercept 50 mg weekly for 24 weeks and observed maintenance of euglycemic state of patients with lower levels of plasma insulin, showing increased insulin sensitivity.

Bonilla *et al.*, as well as Wambier *et al.*, reported the case of a patient with type 2 diabetes who had severe hypoglycemia after initiation of therapy with etanercept and return to normal levels after its discontinuation.^26,27^.^26,27^

There is also a case report of a patient with rheumatoid arthritis and controlled diabetes mellitus that, after three weeks of initiating therapy with etanercept at a dose of 50 mg weekly, began to present unstable diabetes, with two episodes of severe hypoglycemia, separated by at least two days. Discontinuation brought stability to the treatment of diabetes and the introduction of a new anti-TNF-α (infliximab) did not cause the same problem. ^24^

#### Adalimumab

As in the case of infliximab, there is no data analyzing the relation between the use of adalimumab and the change in insulin sensitivity or in glucose levels in patients with psoriasis.^16^

In patients with rheumatoid arthritis, the study of nine cases of patients receiving adalimumab revealed that, although a decrease in inflammatory burden occurred, there was no change in insulin resistance.^28^

#### Ustekinumab

A randomized, placebo-controlled study evaluating the efficacy of ustekinumab within 24 weeks of use did not observe any changes in fasting glucose levels.^29^ Papp *et al.*, in a randomized, placebo-controlled, double-blind study, called PHOENIX 2, evaluated the effectiveness of the medication up to 52 weeks and, similarly, found no changes in fasting glucose.^30^ It is noteworthy that the inclusion criteria for these studies is not quite detailed and there may have been selection bias, decreasing the chance of patients with altered glycemia were initially enrolled for the study.

We still lack randomized, controlled studies, specifically designed for psoriatic patients and that have sufficient statistical power to determine the real effect of immunobiologicals on blood glycemic levels, peripheral resistance and development of DM in psoriatic patients previously without comorbidities. However, the fact that there are many reports of hypoglycemia after using immunobiologicals seems to show a regulatory role of these drugs on the endocrine system still to be clarified.

### 1b. CENTRAL OBESITY

Obesity is associated with higher levels of chronic inflammation and has a central role in the development of metabolic syndrome, appearing to precede other components of the syndrome. ^10^

Metabolic syndrome and obesity occur commonly in patients with psoriatic arthritis and adversely affect the disease activity and response to therapy.^15^

Al-Mutairi *et al.* showed that the body weight reduction in obese patients with psoriasis receiving immunobiological therapy can increase the effectiveness of the drug, and Solomon *et al.* showed that the weight loss (greater than or equal to 5% of the initial weight) regardless of the type of diet is associated with a higher success rate in achieving control of disease activity in patients with overweight or obesity with psoriatic arthritis treated with anti-TNF-α.^31,32^

#### Infliximab

Considerable increase in weight and body mass index (BMI) with the use of infliximab was evidenced by Gisondi *et al.*, and the relative risk of developing increased five kilograms would be 4.3 compared with that observed in the control group, which used methotrexate (MTX).^33^ In another study, it was observed increased weight and BMI until the 46th week of treatment with infliximab, reaching plateau until the 78th week when, gradually, there was loss of weight gain, but no reduction in BMI.^34,35^

Weight gain in patients using this immunobiological drug occurs in the first months and is higher among patients with normal BMI than among those previously obese or overweight.^35^

Similar results were observed in patients with psoriatic arthritis. In the patients with rheumatoid arthritis, the weight increase was insignificant.^16^

The reason why there is weight gain in patients who are on anti-TNF-α agents is not known. ^36^

#### Etanercept

Studies have shown increased weight and BMI with the use of etanercept, as has been described with infliximab. Mean weight gain of 1.5 kg and an increase in BMI of 0.5 kg/m^2^ in the 24th week in retrospective cohort, as well as increasing weight gain up to the 48th week, were observed, particularly in thin patients. ^33,34^

Similar results were also observed in patients with psoriatic arthritis. In patients with rheumatoid arthritis, the increase was slightly significant and entirely based on lean body mass.^16^

#### Adalimumab

The same study of Saraceno *et al.*, evaluating use of infliximab and weight gain, also assessed the use of adalimumab and weight gain, reaching similar results. Mean weight gain in the 24th week was 2.23 kg, being held until the 78th week, when there was a gradual weight loss.^34^

No studies involving the use of adalimumab and weight gain in patients with psoriatic arthritis and rheumatoid arthritis were found. ^16^

#### Ustekinumab

There are reports of increased weight or BMI and use of ustekinumab in patients with psoriasis, rheumatoid arthritis or psoriatic arthritis.

Mean weight of psoriatic patients tends to be higher than those of patients with rheumatoid arthritis, which can be explained by rheumatoid cachexia.^37^ In patients with rheumatoid arthritis, even though they have insulin resistance or other components of the metabolic syndrome, the risk for weight gain with the use of biological drugs is counterbalanced by cachexia induced by TNF.^38,39^

In patients with psoriasis, a large proportion starts treatment with anti-TNF-α with high BMI, without compensation, because TNF does not induce in these patients a "psoriatic cachexia". Thus, the weight gain emerging from the use of anti-TNFs should be thoroughly evaluated and can even be deleterious because it leads to increased cardiovascular risk. This anabolic effect does not occur in patients using ustekinumab, which is an advantage of this drug in obese patients.^36^ If this weight gain is balanced by a decrease in the inflammatory burden also observed in the treatment with anti-TNF-α this is still not known and further studies are necessary. ^37^

### 1c. ATHEROGENIC DYSLIPIDEMIA

#### Infliximab

Few studies have evaluated changes in serum lipids after the use of infliximab in psoriasis patients. In a retrospective cohort study, Gisondi *et al.* found no changes in total cholesterol or in triglycerides after 24 weeks of infliximab. ^33^ Saraceno *et al.*, in a case-control retrospective study, which compared the blood lipids of patients using infliximab with a control group that was in use of methotrexate and efalizumab, observed no significant changes in total cholesterol and its fractions and in triglycerides in a period of 48 weeks.^34^

Studies with patients with psoriatic arthritis are conflicting. While some showed a pro-atherogenic modulation of infliximab after 6 weeks of use, with reduced HDL cholesterol and increased triglycerides, others showed a consistent increase in HDL cholesterol from 4 to 24 weeks after the use of infliximab, without changes in LDL cholesterol or in triglycerides.^16^

In patients with rheumatoid arthritis, however, trials show antiatherogenic modification of blood lipids in the short term and a modulation towards an atherogenic profile in the long term.^40,41^ Most studies that evaluated patients for up to one year show an increase in HDL cholesterol and total cholesterol.^41^ After one year, there is a tendency for HDL and total cholesterol levels to return to baseline levels or below these, with maintenance or increase of LDL cholesterol, total cholesterol and triglycerides.^40^ One study, however, showed an increase in adiponectin, with anti-inflammatory activity, and modulation of the lipid profile for an anti-atherogenic model after one year of treatment with infliximab.^42^

Van Halm *et al.*, evaluating the action of infliximab in patients with ankylosing spondylitis, reported a deterioration of the lipid profile during the use of infliximab only when there is no control of the inflammatory burden, evidenced by the activity of the disease.^43^

#### Etanercept

It seems to be no deleterious influence of the drug on lipid profile of patients with psoriasis according to the studies, although none of these has been specifically designed for the observation of this event, but rather to determine increase of BMI and insulin tolerance in psoriatic patients.^33,34,44^

A retrospective cohort study published in 2011 evaluated 45 patients with plaque psoriasis, moderate to severe, receiving etanercept, and found that there was no statistically significant change in the lipid profile of patients in 24 weeks of use.^45^

In patients with psoriatic arthritis, there is also no indication that there is a change in blood levels of lipids in the long term.^34^

As in psoriasis, there is little evidence on the influence of etanercept in blood lipids in patients with rheumatoid arthritis. A study with more than 24 weeks of duration showed increase in total cholesterol, especially of anti-atherogenic HDL cholesterol, with no increase in LDL or triglycerides.^46^ Studies with less than 24 weeks does not support this finding, showing no lipid alterations in these patients. ^47^

An observational cohort in 2010, assessing 292 patients with rheumatoid arthritis, determined that the use of medication for a year would ensure a decrease in the ratio of ApoB molecules (found on the surface of LDL and VLDL-cholesterol molecules) and Apo-A1 (found in surface of the HDL-cholesterol molecule). Furthermore, the study showed that the greater the inflammatory burden measured by the disease activity, the greater the ApoB/Apo-A1. Therefore, the less chance of modifying the lipid profile for an anti-atherogenic type.^48^

#### Adalimumab

A single study, which examined the levels of blood lipids up to 48 weeks, showed no significant changes despite the increase in BMI and in body weight observed in psoriatic patients analyzed.^34^

In patients with psoriatic arthritis, despite a reported case of a significant increase in lipid levels, there seems to be no change in serum lipids during the use of adalimumab.^34,49^

In rheumatoid arthritis patients, the results are contradictory. A study that evaluated patients treated with adalimumab for only 14 days, compared with control group, showed a significant increase in HDL cholesterol.^40^ At 14 weeks, however, Soubrier *et al.* did not observe changes in the lipid profile. ^47^

#### Ustekinumab

There are no data specifically relating use of ustekinumab to lipid alterations. However, during efficacy studies on long-term use of the medication, consistent changes were not reported. ^30^

It is still unclear the role of biological drugs in the modulation of plasma lipids in psoriatic patients and their consequences on comorbidities, specifically on cardiac risk. However, it is likely that there are differences between the effects of each immunobiological as well as differences in the effect of drugs on different patients. Moreover, as regards Pollono *et al.* in a systematic review on the topic, perhaps more important is the effect of immunobiologicals on the inflammatory burden and their consequences on comorbidities than the lipid profile itself.^50^ This is also demonstrated by the evidence that the lipid profile of patients improve with decreased inflammatory burden, measured by disease activity. ^48^

### 1d. SYSTEMIC ARTERIAL HYPERTENSION

Patients with psoriasis present changes in the renin-angiotensin-aldosterone system. Various publications, including a meta-analysis, have shown an increased prevalence of hypertension among patients with psoriasis and this has been described as being more difficult to control.^10,51^

#### Infliximab

In individuals with rheumatoid arthritis and normotensive, it was observed that the use of infliximab can reduce blood pressure levels, especially during the day, and this may be related to a reduction in sympathetic activity, mediated by reduced serum levels of norepinephrine observed.^52^ To date, there are no references on the impact of the use of this immunobiological on pressure levels of individuals with psoriasis, normotensive or hypertensive subjects.

#### Etanercept

Infusion of etanercept in experimental animals with spontaneous hypertension showed to improve the balance between neurotransmitters and pro and anti-inflammatory cytokines in the paraventricular nucleus of the hypothalamus, reducing disease progression and cardiac ventricular hypertrophy.^53^ Its effect in hypertensive humans has not been studied.

#### Adalimumab

The clinical response of patients with psoriasis treated with adalimumab, measured by PASI-50 and PASI-75, was not affected by the diagnosis of hypertension in a study with 144 individuals with psoriasis and psoriatic arthritis.^54^ References on the evolution of hypertension in patients with psoriasis during or after treatment with adalimumab were not found.

#### Ustekinumab

There are no data on the effects of this immunobiological on pressure levels in both animals and humans, healthy subjects or patients with psoriasis, psoriatic arthritis and/or hypertension.

Animal models seem to indicate that changes in neurotransmitters and balance of inflammatory cytokines in the hypothalamus may be related to a dysfunction of the renin-angiotensin-aldosterone system, which would be responsible for the higher prevalence of hypertension in patients with psoriasis. The action of TNF-α in this area could rebalance these substances and promote a reduction in blood pressure and progression of hypertension. However, further studies in humans are needed to confirm this association. The anti-IL 12/23 agents still need to have their influence on blood pressure levels studied.

## 2. CARDIOVASCULAR RISK AND PSORIASIS

It is known that patients with psoriasis are at increased risk of cardiovascular disease, especially those with severe disease at an early age.^55^ Rose *et al.* compared the vascular inflammation in patients with psoriasis (n=10) and psoriatic arthritis (n=5) with healthy subjects (n=10) using fluorodeoxyglucose positron emission/ computed tomography and found high regional aortic vascular inflammation in patients with psoriasis and psoriatic arthritis compared with healthy individuals, reinforcing the previous finding of premature atherosclerosis in these patients.^56^

The inflammatory response observed in psoriasis leads to insulin resistance, oxidative stress, endothelial dysfunction, and atherosclerosis development, which culminates with acute myocardial infarction or cardiovascular acidents.^15^ Studies suggest that psoriasis may be an independent risk factor for premature cardiovascular events. ^57^

It is likely, at least theoretically, that the inflammatory events that are perpetuated into the bloodstream in psoriasis lesions influence the appearance of vascular endothelium lesions and the development of atherosclerosis. From this theoretical situation also arises the possibility that, diminishing the inflammatory burden, the risk of onset of cardiovascular disease also diminishes. ^58^

On the other hand, it is well known the FDA (Food and Drug Administration) alert for the use of immunobiologicals in patients with severe heart failure, leading to considering that these drugs can cause greater damage than potential cardiovascular benefits.^59^

In patients with heart failure, TNF-α levels are elevated and associated with the severity of clinical signs and symptoms.^60,61^ Experimental models of heart failure suggest that anti-TNF-α could enhance ventricular dysfunction, however a large clinical trial evaluated etanercept in the treatment of congestive heart failure and showed no benefit, while another trial reported worsening of heart failure in patients with moderate to severe chronic disease receiving high dose of infliximab.^56,62,63^ Consequently, the presence of severe heart failure continues to be a contraindication to the use of TNF-α inhibitors. A recent study comparing 8,656 new users of non-biologic therapies with 11,587 new users of TNF-α inhibitors showed that TNF-α inhibitors were not associated with an increased risk of hospitalization for heart failure. ^64^

In efficacy and safety studies of immunobiologicals, when the occurrence of cardiovascular adverse events is analyzed, serious cardiovascular events are usually included - MACE (Major Adverse Cardiovascular Events) - such as acute myocardial infarction, stroke and death from cardiovascular disease.

A potential cause of problems when analyzing the effects of antipsoriatic therapies on comorbidities is the relation between anti-IL-12/23 immunobiologicals and possible serious cardiovascular events, leading to discontinuation of study with briakinumab and considerable concern in the prescription of ustekinumab.^65^

So far, there are only studies that evaluated the association between use of immunobiologicals and incidence of cardiovascular events in patients with psoriasis.^65-67^ There are no studies specifically designed to assess the impact of therapy on cardiovascular risk conferred by psoriasis.

A pooled data collected from four previous phase II and III studies (PHOENIX I and II, ACCEPT and Krueger *et al.* NEJM 2007) performed with ustekinumab had the objective to determine drug efficacy and safety.^66^ The study, in the model of a meta-analysis, showed that there is an increase in the absolute number of MACE in the treated group compared with the placebo group, but this difference, corrected and compared with data from population matched for age and gender was not significant, determined by a number of MACE in patients using ustekinumab of 0.44 patient/year. Although it was not the primary objective of the study, the authors also concluded that there was no benefit of ustekinumab on the incidence of serious cardiovascular events. ^66^

In a meta-analysis published in 2011, Ryan *et al.* evaluated the association between biological therapy for cutaneous psoriasis and cardiovascular events. The study selected 22 randomized, double-blind, placebo-controlled trials, with the specific objective to assess, among other things, the development of MACE. The total number of patients evaluated in the meta-analysis was 10,183. Although 11 patients presented MACE in the treated group compared with one patient in the control group, the corrected data showed no statistical significance. Interestingly, when the biological drugs were divided between anti-TNF-α and anti-IL-12/23, one patient presented MACE in the group receiving anti-TNF-α compared with one patient in the placebo group, while 10 patients presented MACE in the group receiving anti-IL12/23 and none in the placebo group. ^65^

Therefore, there seems to be an increased risk, but with little or no significance, especially with the anti-IL12/23 medication. Low absolute numbers of MACE in patients in use of anti-TNF may also be explained by the known contraindication of its use in patients with severe congestive heart failure, which prevents its prescription in patients with increased cardiovascular risk.

Several recent publications have suggested that TNF inhibitors have a beneficial impact on cardiovascular risk. ^64,68,69^

In a meta-analysis published in 2011, comprising 20 previous publications, Westlake *et al.* summarized the potential effects of anti-TNF-α in patients with psoriatic arthritis not only on MACE (including heart failure), but also on the risk of developing cardiovascular disease again. The authors did not observe a higher incidence of heart failure or worsening of pre-existing disease, and there was no increase in the number of MACE, either absolute or corrected. In fact, the pooled results showed a potential cardioprotective effect of anti-TNF-α, but not as efficient as that observed with the use of methotrexate. This difference becomes more evident when analyzing data of patients in use of anti-TNFs in monotherapy compared with those who use a combination of anti-TNF-α and methotrexate. ^70^

In the same year, another study evaluated the association between anti-TNF-α therapy in rheumatoid arthritis and cardiovascular events, getting different relative risks (RR) among cohort studies and randomized controlled trials. When cohort studies were analyzed, TNF inhibitors were associated with reduced risk of all cardiovascular events (pooled and adjusted RR 0.46, 95% CI 0.28 to 0.77), myocardial infarction (pooled and adjusted RR 0.81 95% CI 0.68 to 0.96) and stroke (pooled and adjusted RR 0.69, 95% CI 0.53 to 0.89). The meta-analysis of randomized clinical trials also estimated lower risk, but this was not statistically significant.^69^

In a retrospective cohort study of 8,845 patients, the use of anti-TNF or oral agents/phototherapy was associated with reduced risk of acute myocardial infarction compared with patients treated with topical agents, which leads us to believe that systemic treatment of psoriasis reduces the inflammatory reaction. ^71^

Study involving 2,400 patients with severe psoriasis showed that patients treated with immunobiological agents or MTX showed low cardiovascular event rates compared with patients treated with other therapies. Among immunobiological agents, treatment with etanercept showed significant reduction of cardiovascular risk markers. ^72^

A recent meta-analysis and systematic review evaluated the relation between the use of anti-TNFs, MTX, non-steroidal anti-inflammatory drugs and corticosteroids, and the presence of cardiovascular events in patients with rheumatoid arthritis (28 studies, 236,525 patients), psoriatic arthritis and psoriasis (6 studies, 220,209 patients). In rheumatoid arthritis, the use of anti-TNF was associated with reduced risk of cardiovascular events (RR 0.7, 95% CI 0.54 to 0.9; p<0.005), acute myocardial infarction (AMI), stroke and MACE. The study found no significant effect regarding heart failure. A beneficial association of MTX with cardiovascular risk reduction was found (RR 0.72, 95% CI 0.57 to 0.91; p=0.007). However, MTX was not associated with decreased risk of stroke and MACE, despite the tendency to decrease the risk of heart failure. This may be due to the low number of events included. Regarding the psoriatic arthritis and/or psoriasis, data were sufficient only to evaluate the effect of systemic therapy compared with not using therapy or using topical therapy. Systemic therapy was associated, significantly, with decreased risk of cardiovascular events (RR 0.75, 95% CI 0.63 to 0.91; p=0.003). ^67^

There are important limitations of studies assessing cardiovascular risk and immunobiologicals. In clinical trials, patients with preexisting cardiovascular disease are excluded. Thus, it is not possible to examine whether there is influence on preexisting conditions. Moreover, they are extensively evaluated in the cardiovascular point of view, which may not be possible in routine of a dermatology clinic due to the high costs. This assertion is supported by meta-analysis that evaluated the clinical trials of efficacy and safety of ustekinumab and showed a large number of psoriatic patients who were not diagnosed but were treated in the cardiovascular point of view compared with the placebo group, which may have contributed to the higher numbers of MACE in the treated group. This shows the risk of comparing different populations, unpaired, for the actual cardiovascular risk.^73^

Another limiting factor of the studies is a possible selection bias. This is true as much as patients with severe psoriasis with high PASI, BSA (Body Surface Area) or PGA (Physician's Global Assessment), in which it is known that the risk of cardiovascular disease is higher, and who are preferably chosen for the clinical trials. This fact is even more important because it is influenced for a bias, either in severity score observed by doctors, or in the DLQI (Dermatology Life Quality Index) observed by patients. In meta-analysis, different studies use different ways to measure the severity of psoriasis and these methods are not always comparable. In the case of the DLQI, the bias is even more serious because it is dependent on the patient's assessment of their body image, which does not always reflect high PASI and hence a greater degree of systemic inflammation. So there is the risk of patients with high PASI be subtreated or that DLQI be overrated as a method of choice for the treatment with biological drugs.

In conclusion, there is a consistent theoretical rationale that impels the use of medications that decrease the inflammatory psoriatic burden caused by the disease and thus attenuate the cardiovascular risk. But to date, there is not enough consolidated data, especially regarding the use of immunobiologicals to cutaneous psoriasis, which authorize the use of these medications with the aim to reducing the cardiovascular risk of patients. In fact, in those patients who tolerate, the associated use of methotrexate are more likely to cause protection to the cardiovascular system than the use of isolated immunobiological. Moreover, it is important to remember that the other variables included in the criteria of Framingham for cardiovascular risk may have greater impact on the life expectancy of patients than psoriatic disease alone.

## 3. NONALCOHOLIC STEATOHEPATITIS AND PSORIASIS

Nonalcoholic steatohepatitis (NASH) is insidious fatty infiltration of the liver and is recognized as a consequence of obesity, especially associated with metabolic syndrome. It is the leading cause of altered liver function tests in developed countries.^72^ In psoriatic patients, NASH affects 47% of patients with chronic plaque psoriasis and 27% of the control non psoriatic population, matched by gender, age and BMI, and is more pronounced in more severe psoriasis and with longer disease duration. The steatotic liver produces proinflammatory and pro-atherogenic cytokines, basically C-reactive protein (CRP) and interleukin-6 (IL-6), and decreases the production of adiponectin that, unlike the leptin, has anti-inflammatory activity. This increases the risk of severe disease by increased inflammatory burden, increasing cardiovascular risk.^74^

There are no controlled studies evaluating the impact of treatment with any of immunobiologicals on NASH.

Study of 89 patients with moderate to severe plaque psoriasis diagnosed with metabolic syndrome and NASH receiving therapy with etanercept or PUVA after 24 weeks of treatment showed a significant decrease in the levels of ALT, AST, CRP and insulin in the group that used the immunobiological.^75^

Studies in non psoriatic murine animal model, fed with high fat diet, showed the possibility of infliximab reverse steatosis triggered by poor diet.^76^ Another study in an animal model, with mice deficient in choline and with methionine-induced hepatitis, showed that infliximab (as anti-TNF-α drugs prototype) decreased fat infiltration and hepatic fibrosis.^77^ A case report of a patient with rheumatoid arthritis using adalimumab demonstrated reduction of steatosis and improvement of liver function tests.^78^

Patients with psoriatic arthritis treated with anti-TNF-α and associated MTX presented a lower risk of liver fibrosis than those treated with MTX alone. ^15^

It is not possible to state that immunobiologicals can alter the course of NASH due to the lack of studies specifically designed for this purpose. The evidence in animal models and theoretical evidence allow, at least, suggesting that the action of these drugs on fat infiltration and inflammation in the liver of patients with psoriasis is possible. However, if this is caused by the direct action of the medication or if it is caused by the control of systemic inflammation, as measured by decreased serum levels of proinflammatory cytokines (as IL-6 and CRP), it is still not possible to know.

## CONCLUSION

To date, analysis of evidence in the literature reveals little about a definitive role of immunobiologicals on the metabolic syndrome in patients with psoriasis and psoriatic arthritis.

However, if we extrapolate the data found in studies with patients with rheumatoid arthritis, we can observe a long-term improvement trend in glycemia and insulin sensitivity in patients treated with immunobiologicals, improving the clinical outcome of patients. On the other hand, the analysis of the data generated in patients with rheumatoid arthritis shows little effect or even worsening of the lipid profile, with the possible exception in the case of etanercept, but still needing confirmation. Thus, a possible beneficial effect of immunobiologicals on glycemia can eventually compensate a deleterious effect, or even a lack of effect, on plasma lipids.

We highlight the importance of the inflammatory burden on the clinical outcome of patients, not only on the activity of the disease, but also on comorbidities. In this sense, systemic treatments, either classical or immunobiological, may have a fundamental role to effectively control the inflammatory burden on psoriatic patients, decreasing the chance of comorbidities, more specifically of metabolic syndrome.
